# BioIMAX: A Web 2.0 approach for easy exploratory and collaborative access to multivariate bioimage data

**DOI:** 10.1186/1471-2105-12-297

**Published:** 2011-07-21

**Authors:** Christian Loyek, Nasir M Rajpoot, Michael Khan, Tim W Nattkemper

**Affiliations:** 1 Biodata Mining Group, Faculty of Technology, Bielefeld University, Universitätsstraße 25, 33615 Bielefeld, Germany; 2 Computational Biology and Bioimaging (COMBI) Group, Department of Computer Science, University of Warwick, Coventry CV4 7AL, UK; 3 Biomedical Research Institute, School of Life Sciences, Gibbet Hill Road, Coventry CV4 7AL, UK

## Abstract

**Background:**

Innovations in biological and biomedical imaging produce complex high-content and multivariate image data. For decision-making and generation of hypotheses, scientists need novel information technology tools that enable them to visually explore and analyze the data and to discuss and communicate results or findings with collaborating experts from various places.

**Results:**

In this paper, we present a novel Web2.0 approach, *BioIMAX*, for the collaborative exploration and analysis of multivariate image data by combining the webs collaboration and distribution architecture with the interface interactivity and computation power of desktop applications, recently called rich internet application.

**Conclusions:**

*BioIMAX *allows scientists to discuss and share data or results with collaborating experts and to visualize, annotate, and explore multivariate image data within one web-based platform from any location via a standard web browser requiring only a username and a password. *BioIMAX *can be accessed at http://ani.cebitec.uni-bielefeld.de/BioIMAX with the username "test" and the password "test1" for testing purposes.

## Background

In the last two decades, innovative biological and biomedical imaging technologies greatly improved our understanding of structures of molecules, cells and tissue and increasingly have become high-throughput and high-content applications. These new technologies produce a deluge of complex bioimage and meta data. Biologists and clinicians are faced with the difficult task of discovering and extracting knowledge based on the newly acquired data to generate hypotheses or diagnoses. Therefore, they need software tools for data analysis, and this poses new challenges to the image computing community in consequence. In addition to the existing vital and expanding field of image processing, novel information technology approaches have to be developed to extract, compare, search and manage the data and the knowledge, e.g. semantic annotations. As a result, an emerging new engineering area has been evolved, which is called *bioimage informatics *[1]. The key challenge of bioimage informatics is to present the large amount of image data in a way that enables them to be browsed, analyzed, queried or compared with other resources [2].

In the recent literature, first open source bioimage informatics tools have been proposed, which offer functions for processing and analyzing the large amount of complex bioimage data. In [1,2] the most popular tools have been summarized and discussed concerning different aspects, i.e. image analysis, data management and retrieval or data visualization and evaluation. The variety of existing tools can be divided mainly into three different categories: general image analysis tools, single purpose tools and analysis platforms. General image analysis tools like ImageJ [3,4] or ITK [5,6] can be applied for a large variety of image processing tasks such as registration, filtering, thresholding and segmentation. Single purpose tools focus on special biological or biomedical problems as well as on data from specific imaging techniques. As a prominent example the CellProfiler system [7,8] has been developed as a desktop application to automatically screen cellular images to gather information such as number of cells, cell sizes or other morphological features of cells. Recent developments in the field of bioimage informatics tend towards a third category that we refer to as analysis platforms. The idea of analysis platforms is to store and organize large amounts of image data in a central repository on remote server architectures, accessible either via local clients or through web based interfaces, in order to allow fast and efficient data retrieval in a distributed environment. In addition to data management issues, those platforms can include selected methods for data visualization, annotation and analysis. One of the first tools published in this context is OME (Open Microscopy Environment) [9]. OME provides a client-server platform (OMERO) [10] for visualization, management and annotation of highly structured scientific image data. OMERO is installed locally on users workstation or on a server in large research departments or laboratories. Bisque [11] is a recently introduced powerful tool, which provides a web-based platform for image management, sharing and annotation. It includes web-based analysis routines, e.g., an automatic 3D nuclei detector or a microtubule tracker, as well as external desktop based analysis tools using the Bisque data repository. In addition, Bisque provides an interface for custom analysis modules, which allows one to develop novel web or desktop applications and to connect those routines to the Bisque system.

Bioimage informatics is a young but highly active research field and has already contributed to the solution of many biological questions. Most of the proposed tools are focussed on particular well defined biological problems or imaging modalities and provide specially adapted solutions to solve these problems. However, in many cases the analysis goal is vague and little *a priori *knowledge is available for the underlying data. Thus, it is not clear in advance, which analysis strategy or method should be used. This applies in particular to new imaging modalities such as MALDI imaging [12], i.e. if the images were acquired with new imaging techniques or the biological sample was imaged for the first time or to images where the valuable information is not directly accessible, which is especially the case regarding *multivariate images *(MVI). Multivariate imaging strategies aim at gathering images that reflect different (molecular) characteristics of the sample [13-16]. The resulting image stack contains *n *different channels, where each spatial location is associated to *n *different signals, referred to as the signal domain. In combination with the spatial domain, the signal domain is of particular biomedical value, so researchers need an initial exploratory access to the image information in a fast and intuitive way to aid the process of early steps in analysis and knowledge discovery, i.e. forming a mental model for the data and developing hypotheses. Furthermore, in this analysis context, it is of particular importance to provide tools for discussion with the collaborating research community about hypotheses, results, or findings. Nowadays, collaboration is more important than ever before in life science projects [17]. Different analysis aspects often call for expert knowledge from different fields like medicine, biology, pharmaceutics, statistics, physics or computer science. A successful (cross-discipline) collaboration is often impeded, since the involved researchers are usually spread across several research institutes.

In view of above observations and arguments, the following features must be owned by a novel platform for bioimage analysis, which are not covered by existing bioimage informatics solutions. First, it has to provide tools for the visual exploration of MVIs. Methods from the fields of exploratory data analysis (EDA), visual datamining (VDM) or information visualization are ideally suited to cope with such image analysis problems. Here, the basic idea is to present the data in some visual form, allowing the human to directly interact with the data by adjusting and manipulating visual data displays, so that visualization is rather becoming an analysis and exploration tool than an end product of automatic analysis [18]. Second, scientists need facilities to exchange information with other scientists, i.e. sharing scientific data and image related information, e.g. by free graphical and textual annotations, which might be linked directly to image regions and coordinates. Although desktop solutions such as CellProfiler or OMERO provide some interactive data displays, they lack collaboration abilities for geographically distributed scientists. In contrast, web-based bioimage analysis solutions like Bisque offer far better collaboration and data sharing capabilities, since recently the web is getting more collaborative and user-shaped (effects that are referred to as Web2.0), but they only include rudimentary web-based data visualization and interactivity facilities. In contrast to the aforementioned solutions, we propose a free, fully web-based software approach, called *BioIMAX *(**Bio**Image **M**ining, **A**nalysis and e**X**ploration), developed to augment both an easy initial exploratory access to a large variety of complex multivariate image data and a convenient collaboration of geographically distributed scientists via the web. This focus on quick collaborative visual exploration and analysis of a large variety of bioimages ranging from spectral data to multi-tag fluorescence images.

*BioIMAX *was developed as a Rich Internet Application (RIA). RIAs are web applications whose performance and look-and-feel is comparable to standard desktop applications, offering powerful graphical visualization and interaction capabilities. RIAs are running in a web browser allowing for platform independency and avoiding additional installation and maintanence costs. The application of RIAs as part of the change of the World Wide Web towards Web2.0, recently called *Social Media*, is becoming increasingly important. *BioIMAX *is the attempt to explore the potential of these social network technologies in the context of bioimage analysis by combining the webs lightweight collaboration and distribution architecture with the interface interactivity and computation power of desktop applications. Such internet-based scientific collaboration is called Science2.0 [19,20] and is an active research area for years, e.g. in the field of health care and medical or clinical research [21]. With *BioIMAX*, several types of (multivariate) image data can be uploaded and organized in personalized projects through a simple web-based interface. This allows a rapid data search and retrieval of one's own datasets and supports easy sharing of data with other collaborating researchers by inviting them to join created projects. Furthermore, project members are able to annotate, discuss and comment on specific image regions with the *BioIMAX Labeler*, a graphical and textual annotation tool. In order to initially explore multivariate image data, the *BioIMAX VisToolBox *provides general methods to get visual access to the *n*-dimensional signal domain of MVIs, e.g. by visualizing each two-channel combination with scatter plots or by a parallel coordinates plot to compare large numbers of variables in one plot.

## Implementation

As previously mentioned, *BioIMAX *software was designed as a Rich Internet Application. The usage of RIAs has several advantages, which meet the necessary requirements for the development of a system like *BioIMAX*. In contrast to conventional thin-client web applications, RIAs provide a richer and more complex graphical interface, resembling desktop applications' interface interactivity and computation power. The RIA technology improves the efficiency of web applications by moving part of the computation, interaction and presentation to the client, thereby lowering the amount and frequency of client-server traffic considerably and permitting asynchronous client-server communication. As a result, the usability of web applications will be improved, annoying installation routines will be avoided and the software is accessible from any location.

The *BioIMAX *client side was developed with *Adobe Flex *[22], which is an open-source framework for building expressive web applications. RIAs developed with Adobe Flex deploy consistently on all major browsers and operating systems by leveraging the *Adobe Flash Player*. The Flash Player is a proprietary web browser plug-in, which may be considered as a drawback. However, the Adobe Flex SDK is open source and the Flash Player is available for free download. The Adobe Flex framework provides a comprehensive collection of predefined standard components, which allows developers to rapidly create visually appealing web application prototypes that can be customized and extended depending on their needs. Since RIAs usually provide a richer graphical user interface and a considerable amount of application logic, this could lead to longer loading time when initially starting the application and to increased computational cost of the client workstation than with conventional HTML-based web applications such as the Bisque system. In order to efficiently and consistently manage the data collected, *MySQL *[23] is used as a relational database management system. The communication between the Flex client and the server-side database is realized by using *AMFPHP *[24]. It is one of the fastest client server communication protocols available to Flash Player developers, because communication is serialized into a binary format that is generally more compact than other complex types such as XML.

**Figure 1:**
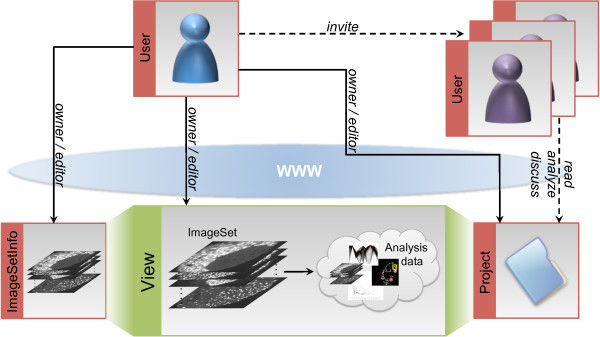
**A schematic diagram, illustrating the structure of the *BioIMAX *data model**. The data model consists of four components: *User*, *ImageSetInfo *, *Project *and *View *. The first three components represents meta information about the respective components and can be referred to as the *meta data category*. In contrast, the purpose of the *View *is to connect the other three components with the actual *analysis data category*, i.e. raw or preprocessed images/MVIs or graphical and textual annotations. The *View *can be considered as the core element of this data model. All analysis results are interpreted as a view of the raw ImageSet/MVI data, allowing a simple and flexible integration of new tools and types of analysis data. In addition, the *BioIMAX *data model includes user rights management, associating Views, ImageSets and Projects to the owner, who has uploaded or created them and allows the owner to invite other users to join projects. After joining, other users are able to read project-related data and to provide new data.

*BioIMAX *has to handle several types of data with varying degrees of complexity, which basically can be divided into two categories: (i) *analysis data*, including the raw (multivariate) image data and all kinds of derived data, e.g. preprocessed images or graphical and textual image annotations and (ii) *meta data*, including additional image-related or biological meta knowledge and user-/project-related information. This requires an appropriate data model reflecting the multitude of highly parameterized and interlinked data. The *BioIMAX *data model basically consists of four core components: (1) *User*, (2) *ImageSetInfo *, (3) *Project *and (4) *View *:

(1) The *User *component represents user relevant information and is required for linking the uploaded data to the users. In addition, this component is important for the implementation of a user rights management, allowing one to hide sensitive data from the public.

(2) The *ImageSetInfo *component represents meta information about multivariate images. This includes basic information like image acquisition parameters and biological or clinical meta information, e.g. tumor specifications or anonymized patient records. An ImageSet is equivalent to an MVI, representing a set of *n *single images with linked structure, but formulated in a more general term.

(3) The task of the *Project *component is to collect and organize images and other data within a defined context, e.g. concerning a particular biological or medical question. Other *BioIMAX *members can be invited and linked to a project whose owner wants to setup a group/community of collaborating members (see Figure 1). Members of a project have access to all project-related data and the project component itself manages the permissions to view and analyze this data.

(4) The components of the data model introduced so far mainly refer to the handling of the *meta data category*. The *View *is the central component within the *BioIMAX *data model. It connects the components of the *meta data category *(see (1)-(3)) with the *analysis data category*, i.e. the raw images and its derived data. All kinds of analysis data will be interpreted as a *view *of the raw MVI. Views are characterized by a specific type. For example, a newly uploaded MVI will be identified as an *original view*, whereas a set of graphical annotations will be described by a *label view*. The analysis data associated to a view is managed in an application-specific manner, i.e. data will be stored in an appropriate format or type on the server, depending on the application, which has to load or process the respective data.

The *BioIMAX *data model has a simple but powerful structure, since it is based on just a few essential components, which allow a fast and transparent access to the data with regard to specific *BioIMAX *applications. Due to the *View *concept, an easy and flexible integration of new tools together with its data by *rapid prototyping *is possible, without changing the entire data model for each new application. The basic structure of the *BioIMAX *data model and the relationships of the four different components is shown in Figure 1.

## Results

**Figure 2:**
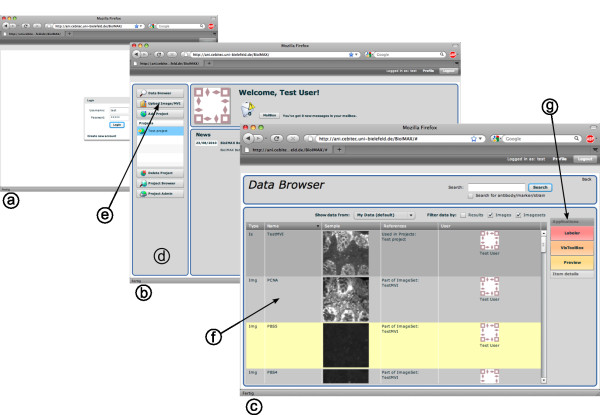
**Screenshots of three interfaces, illustrating the basic steps for using the *BioIMAX *system**. (a) shows the login screen, (b) the start page and (c) the data browser page. After the login procedure with a username and a password, the user is presented with the start page, showing a personalized header, a news box and an initial menu panel (d). The menu panel offers means for basic data handling, e.g. uploading new image data (e) or starting the data browser for searching and browsing the *BioIMAX *database. The data browser displays the search results as items in a datagrid (f) and serves as starting point for further data analysis with previously selected items (g).

In the following, we describe and illustrate important aspects and tools of the *BioIMAX *system. Once a user has been authenticated by a username and password login procedure (see Figure 2(a)), she/he is presented with the *BioIMAX *start page (see Figure 2(b)). The start page is designed in the style of a social media platform, creating a personalized environment. It can be considered as the main page providing access to the various aspects of the *BioIMAX *platform. Through a navigation panel (see Figure 2(d)) users are able to get in contact with other registered users, to upload and organize image data and to start several analysis tasks via the *BioIMAX *data browser.

### Image Upload and Data Sharing

Using the *image upload *interface (see Figure 2(e)), users are able to easily feed MVI data into the systems database. During the upload process users have the option to add descriptive names/tags to each of their image files (e.g. antibody tags). While typing, the system analyzes user keystrokes and suggests predefined tags of an extensive tag list provided by the *BioIMAX *database. In this way the users are encouraged to select one of the predefined tags, in order to keep the namespace of possible tags small, to avoid redundancies and to ensure that images showing the same content get the same tags, so as to simplify the task of searching for particular images for further analysis tasks. These tags are linked directly to the database entry representing the respective image file.

*BioIMAX *also supports sharing of MVIs and analysis results. Therefore, the users can create specific projects, add an arbitrary number of MVIs or results to these projects and invite other *BioIMAX *users to join their projects. This allows project members to quickly access, read and analyze a specific subset of data, e.g. regarding a defined biological or analytical question. This kind of data sharing is the first step towards collaborative work in *BioIMAX*.

Since *BioIMAX *is intended to be an open platform for scientific images freely available to scientists occupied with some kind of MVI analysis, data protection and security is a crucial aspect that should be considered. For this reason, *BioIMAX *provides privilege mechanisms, which ensure that scientific data being confidential remains confidential within *BioIMAX*. Users are able to decide to what extend their data is available to the public. To achieve this, all datasets stored in the *BioIMAX *database are associated with access rights that can be controlled by each user itself. Using projects, data access can be limited to certain individuals, whereas project-related data remains hidden for non-members.

### Querying the database

The *BioIMAX *data browser allows the user to search, browse, extend, modify and manage the image data stored in the *BioIMAX *database (see Figure 2(c)). After starting the data browser page, all user-specific data is retrieved from the server and displayed as *data items *in the sortable browser datagrid (see Figure 2(f)). The search results are *view *instances like image sets, single images and analysis data, e.g. label results. Each datagrid item provides a thumbnail view of the respective images and a descriptive icon for results. Items representing image sets allow the user to rapid eye-balling through the image set by moving the mouse cursor over the thumbnail view from left to right.

The data browser provides several options for searching, filtering or sorting the data stored in the database regarding specific criterions, e.g. it allows the user searching the based on a keyword/tag query, in order to retrieve only those images that are associated to the given keyword/tag.

In addition to visualizing and managing the search results, the *BioIMAX *data browser serves as a starting point for all data exploration tasks. Depending on the semantic type of a selected datagrid item, which corresponds to its view type, the data browser provides several tools that can be started with the selected item. The list of possible tools adapts itself to the type of the selected item, since not all items can be started with all tools.

### Image Viewer

For the purpose of viewing MVIs and analyzing image content, *BioIMAX *tools include a basic image viewer, especially designed for multivariate images. The image viewer loads selected images via the web depending on the data size and the connection strength (e.g., a set of 25 images with 1 MB/image on average takes approximately 25 seconds on average with a maximum connection strength of 16 MBit/sec.) and allows one to scroll through an MVI image by image. The transition from one image to another is performed directly *avoiding *a blank screen between two consecutive images. Avoiding such blank screen is an extremely valuable feature, otherwise the human visual system does not recognize any significant differences between two images, which is referred to as *change blindness *[25]. This feature is not owned by many other tools, since its realization is usually a non-trivial programming exercise. The viewer includes zooming and panning functions for image navigation purposes. Another feature of this image viewer is that zooming and panning is performed not only on the currently displayed image, but in parallel on each image in the MVI. This allows focussing on interesting image regions by zooming and panning and comparing such regions with other images of the MVI without losing orientation.

### Signal analysis of MVI

**Figure 3:**
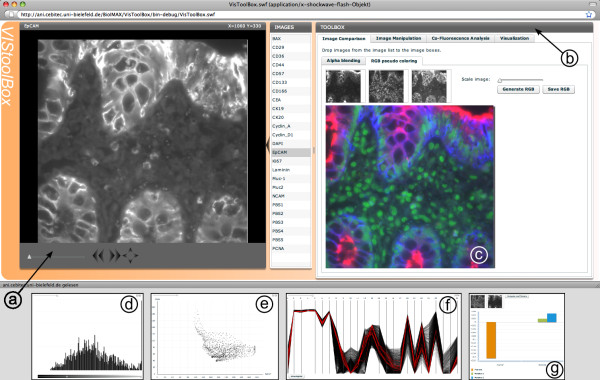
**Screenshot of the *VisToolBox***. This tool provides several methods to explore and analyze the signal domain of multivariate images (MVI). It consists of an image viewer (a) and a panel (b) including methods from the fields of visualization, e.g. creating pseudo color fusion images from selected images (c), co-location analysis and exploratory data analysis (EDA). The small figures below exemplary show further exploration displays: (d) Histogram. (e) Scatter plot. The parallel coordinates plot in (f) depicts the multivariate pixel information of all nine images of the MVI in a compact display. Each vertical compound line represents an unique location within a user-defined image region and shows the corresponding signals of all images at that location. In (g) a co-location study of two images with statistical measurements (Manders' score and Pearson correlation coefficient) is displayed.

The *BioIMAX VisToolBox *(triggered via Figure 2(g)) and illustrated in Figure 3) provides a set of methods to explore and analyze the signal domain of MVIs, i.e. methods from the fields of visualization, co-location analysis and exploratory data analysis (EDA).

#### Image comparison

The *VisToolBox *provides two different methods to compare up to three single images of the MVI simultaneously on a structural/morphological level. The *Alpha blending *method aims at comparing three images while superimposing them as layers and manually adjusting the opacity value of the respective layers by moving the mouse cursor over an opacity triangle. Thus, the user can detect structural differences or similarities between the selected images. The method (*RGB pseudo coloring*) (see Figure 3(c)) generates a pseudo color fusion image from three selected images, by interpreting each image as one color channel in a RGB image. The color of a pixel or region of the resulting RGB image indicates the accumulated amount of signals in the three images.

#### Image Manipulation

In order to filter out irrelevant signals like signals belonging to the background or outliers, the *VisToolBox *includes two interactive histogram dialogs: One representing the relative frequency whereas the other shows the cumulative distribution of image grey values in the currently selected image. The user can manipulate the distributions and a visualization of the result is adapted in real-time in the image viewer.

#### Co-Fluorescence analysis

Here, the user can compare two selected images on a statistical level by calculating (i) the Pearson correlation coefficient or (ii) the Manders' score, which is a frequently used index for co-location studies in fluorescence microscopy [26]. The results are displayed in a bar chart (see Figure 3(g)).

#### Visualization

**Figure 4:**
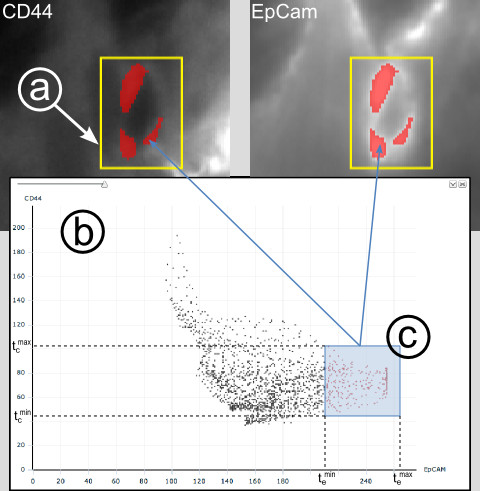
**Interactive exploration of bivariate data from a selected region of interest (ROI) in the image (a)**. For the ROI (a), the user selects two image channels (here the CD44 channel and the EpCam channel) and one tool, e.g. a scatter plot (b) from the visualization tab (see figure 3). The pixel values corresponding to the same location within the ROI are displayed as points in the scatterplot. Selection of points  in the plot (c) triggers highlighting the referring pixels in the image (displayed as red regions superimposing on the original image), with respect to the following criterion: , with Γ escribing the selection of points  in the scatter plot,  and  defines the minimum and maximum of the selection range regarding EpCam values and  is the EpCam value of point . The same applies to the CD44 values, accordingly. This process is often referred to as "gating" or "link-and-brush".

For a more detailed exploration of specific image regions, the user first selects a region of interest (ROI) by drawing a rectangle on the displayed image. All pixels within the ROI will be displayed in a plot chosen by the user, i.e. a histogram, scatter plot or parallel coordinates. Selection of points in one plot triggers highlighting the referring pixels in the image on the left (for detailed description see Figure 4 and additional file 1: Figure 1). This process can also be referred to as "gating" or "link-and-brush" [27].

The *VisToolBox *is developed using standard display elements and their behavior provided by the Adobe Flex framework, e.g., the charting components such as the histogram or the scatter plot displays, and is enriched by customized user interactivity capabilities. Other features such as the image comparison using mouse move events on a triangle or the parallel coordinates plot are implemented from scratch, since these are not covered by the Adobe Flex framework.

### Graphical and semantic image annotation

**Figure 5:**
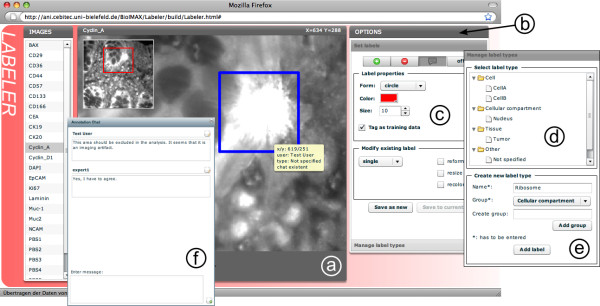
**Screenshot of the *Labeler***. The *Labeler *provides an image viewer (a) including all navigation functions as with the other tools, but is extended with the ability to place arbitrary numbers of graphical objects/labels on single images/channels. A toolbar (b) allows users to adjust and modify several label properties such as geometry, color or size (c) and provides the option to store sets of labels to the database. Single annotations can be characterized by specific semantic types that can be selected from a predefined list of existing types (d) or by adding new user-defined label types and categories (e). In (f) an annotation chat window is shown, illustrating a current state of conversation of two users about the selected label.

The *BioIMAX Labeler *tool allows the user to graphically and semantically annotate image regions in single images/channels of an MVI (see Figure 5). A graphical label is characterized by visual properties, i.e. shape, color, size and position, which can be adjusted by the user at any time. Annotations are placed as graphical objects on a layer belonging to each single image/channel, allowing for easily modifying existing label sets, e.g. delete single labels or change label positions or other properties. A set of labels can be stored into the database by saving all visual properties for each single label and is associated to a new *label view*, which links the label set to the respective MVI. The *Labeler *also provides several options for the user to add comments/questions/semantic annotations to each graphical label and this forms the key feature of this tool.

With the *Labeler *tool we are aiming at two goals. First, users shall be enabled to label interesting image regions associated with *low level semantics*, which can be important in quantification and evaluation tasks. For this purpose, the user has the option to select specific label types from predefined semantic categories, e.g. *Nucleus *from the category *Cellular compartments*. In some cases, the predefined types are not sufficient to describe the semantic of an image region. Therefore, the user can extend the existing "ontology" by creating own categories and types. These types will be associated to each label in the database with a short descriptive tag. Second, chat-like discussions can be linked to image regions to link *high-level semantics *to morphological features. Therefore, the *Labeler *provides a chat window, where several users can communicate about the selected label and the conversation will additionally be stored together with the label. This facilitates Web2.0 style collaborative work on one image, while the stored states of communication content are directly linked to image coordinates/ROIs.

## Discussion

In this paper we presented a Web2.0 approach for the collaborative exploration of multivariate bioimage data. Due to the complexity of such high-content image data, the generation of analysis strategies or specific hypotheses is a difficult task for researchers and is often not straightforward. Thus, methods in the field of exploratory data analysis are very useful for gaining insights into the structural characteristics of the underlying data. By using the *BioIMAX VisToolBox *the user has several options to inspect and visualize various aspects of the multivariate image domain, following Ben Shneidermans information visualization mantra: "Overview first, zoom in and filter, details on demand". The concept of *BioIMAX *does not include predefined automatic analysis workflows regarding a special biological question in the form of a black box model, with image as input and the finalized result as output. With *BioIMAX *the user is directly involved in the knowledge discovery process, while exploring the data space themselves with specific information visualization techniques. This is an important strategy in the field of visual datamining and -exploration [28].

The next important aspect is the collaborative part of *BioIMAX*. Due to a start page interface, which resembles aspects of well known social media environments, users can easily build up small communities by creating projects with the objective to group collaborating members, e.g. experts from different disciplines, together with data regarding specific biological subjects. This allows a clear organization of project relevant data associated with rapid access to the data. Through an internal messaging system, communication by mails is possible and remains within the *BioIMAX *platform, so that no additional email addresses has to be exchanged. Besides the organizational issues, the *BioIMAX Labeler *enables a group of users to discuss and communicate about specific image regions, by labeling regions of interest with graphical annotations and starting a chat conversation about the respective annotation. Such a collaborative annotation tool speeds up and simplifies the communication about image regions and is of great value to avoid misunderstandings.

*BioIMAX *was designed as a Rich Internet Application, which has important advantages over existing bioimage informatics solutions like OMERO or Bisque. The fact, that communication/collaboration and visual exploration is completely performed within one web-based platform requiring only a *username *and a *password *is of great value for both, *BioIMAX *users and developers. Except for the installation of the Flash Player plug-in, which is available for all major browsers, users get rid of cumbersome installation routines of software packages or libraries and have direct access to sophisticated and powerful bioimage analysis and exploration functionalities. Developers are able to keep the *BioIMAX *system up to date and to quickly integrate novel features or tools even as rapid prototypes without adapting *BioIMAX *to the variety of operating systems. Another great advantage of systems like *BioIMAX *is data reproducibility due to data storage in a central data repository. All users work on the same copy of an image, which prevents ambiguity and misinterpretations, e.g. based on different preprocessing steps, while discussing specific aspects of images. This is also an important point for future releases of *BioIMAX*. In addition to the existing *BioIMAX *tools focussed on exploration, visualization and annotation, new tools will be developed, which allow the users to apply specific analysis methods from the fields of image processing, pattern recognition or datamining. The integration of novel tools with extended functionalities in *BioIMAX *is accomplished via a simple standard interface. New tools can be designed and implemented from scratch as stand-alone Flash applications such as the *VisToolBox *or the *Labeler*, which will be integrated into the main *BioIMAX *application using an interface class responsible for exchanging specific parameters and launching the tool in a separate web browser window. Reproducibility from raw data is the first requirement for the realization of such new tools. Users should be able to trace back any result in *BioIMAX *to the original experimental data on which the whole analysis is based. Therefore, each analysis result should come with a "history" file specifying all informations, e.g. which method and parameters were used. For this reason, the *view *concept of *BioIMAX *lays down a framework to integrate and handle such analysis records. Future releases of *BioIMAX *should also provide flexibility to handle data with different file formats, e.g., using standardized file formats such as those the OME group has proposed [29].

In its current state, *BioIMAX *is only available as a single web instance with a central data repository hosted by the authors institute, since it is still an ongoing development process. However, it is available for free use for any scientist willing to apply existing *BioIMAX *functionalities (see additional files 2 and 3 for the demonstration of recent case studies). We will keep all users informed about the recent status of the system and its tools. After a comprehensive testing and debugging phase, we plan to provide both, a stable and documented version of the system as a single web instance as with the current state and as an open source project, which can be downloaded, installed and run locally in laboratory or institute specific environments. However, we believe, that the main benefit of *BioIMAX *lies in a small number of instances used by a large number of researchers, to support the collaborative aspects of research.

## Conclusions

With *BioIMAX *we have demonstrated the potential of Web2.0 technologies for the analysis of complex bioimage data. We have shown, that modern web technologies in the form of RIAs are extremely powerful and allows the development of sophisticated scientific web applications running in the web browser with the computation power of desktop applications. *BioIMAX *exploits this advanced interface interactivity and combines it with the communication and collaboration capabilities offered by the very nature of the Internet. In life science, collaboration between experts of different disciplines located at geographically distributed locations is a crucial part in scientific reasoning, in particular regarding bioimage analysis, and is still a time-consuming and laborious task. Using *BioIMAX*, the collaboration hurdles can considerably be lowered while shifting sophisticated analysis and interactive visual exploration of bioimage data from the users' desktop to the web. Thus, *BioIMAX *permits a faster and easier evaluation and discussion of ideas, hypotheses, data or results via the Internet independently of the users' whereabouts. We believe, that our approach allows new perspectives in the analysis of complex high-content image data, complement to the existing bioimage informatics solutions.

## Competing interests

The authors declare that they have no competing interests.

## Availability and requirements

• **Project name: **BioIMAX

• **Project home page: **http://ani.cebitec.uni-bielefeld.de/BioIMAX

• **Test account: **Login name: "test", Password: "test1"

• **Operating system(s): **Platform independent

• **Programming language: **Adobe Flex 3 Software Development Kit

• **Other requirements: **FlashPlayer 10.1 or higher, tested browsers: Firefox 3.6, Internet Explorer 9, Safari 5.0

## Authors' contributions

The software was conceived, developed and designed by CL. The manuscript was prepared and written by CL and TWN, who supervised the work. NMR has contributed to writing the manuscript. The example image data has been provided by MK and NMR. All authors read and approved the final manuscript.

## Supplementary Material

Additional file 1 **Screenshot of the *VisToolBox***. Click here for file

Additional file 2 **Case study 1**. Click here for file

Additional file 3 **Case study 2**. Click here for file
