# Supplementary figures and images for: BioIMAX: A Web 2.0 approach for easy exploratory and collaborative access to multivariate bioimage data
Source: BMC Bioinformatics. 2011 Jul 21;12:297. doi: 10.1186/1471-2105-12-297 (PMC3161928; doi:10.1186/1471-2105-12-297)

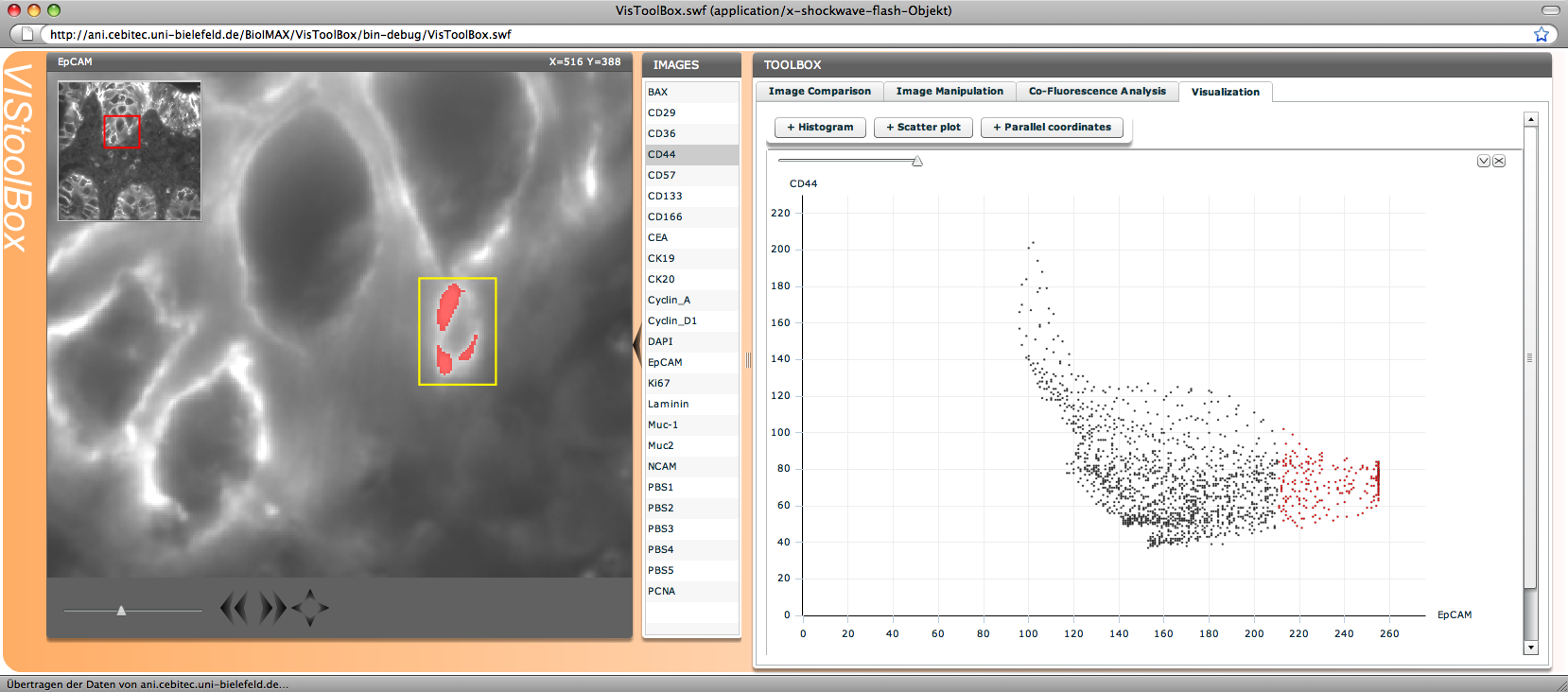

Supplement: Additional file 1 — Screenshot of the VisToolBox. [file 1471-2105-12-297-S1.PNG]
