# Supplementary material for: BioIMAX: A Web 2.0 approach for easy exploratory and collaborative access to multivariate bioimage data
Source: BMC Bioinformatics. 2011 Jul 21;12:297. doi: 10.1186/1471-2105-12-297 (PMC3161928; doi:10.1186/1471-2105-12-297)
Supplement: Additional file 2 — Case study 1. [file 1471-2105-12-297-S2.PDF]

## Case study 1

Studying cell infection grade caused by *Listeria monocytogenes*. Image data has been acquired by a high-content screen using automated microscopy resulting in three channels showing cytoplasm, nuclei, and *Listeria monocytogenes*. The goal of this study is to assess the location and severity of *Listeria monocytogenes* infection. In the following, example screenshots of *BioIMAX* are shown, in order to illustrate its applicability for the exploration of *Listeria monocytogenes* cell invasion.

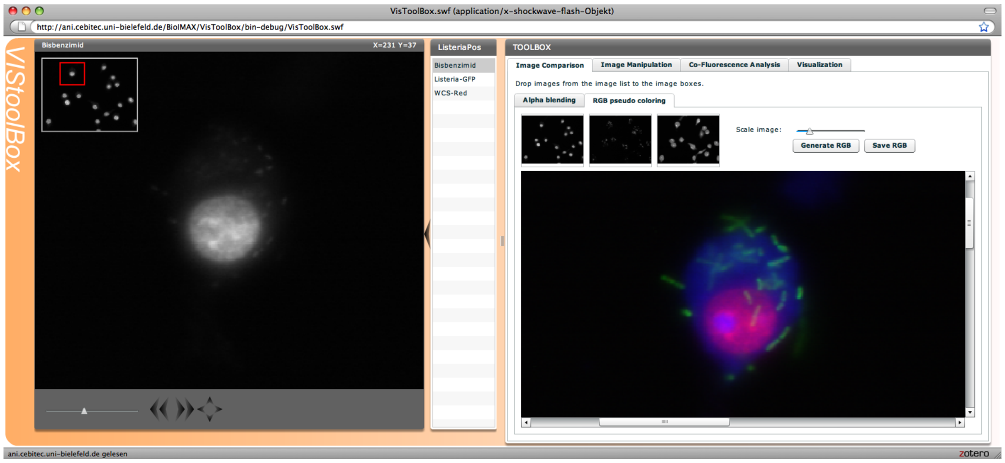


Figure 1: Comparing all images simultaneously with the *RGB pseudo coloring* method. Here, it is possible to get an first qualitative overview about the location and the severity of *Listeria monocytogenes* infection. (Red: nucleus; Blue: cytoplasm; Green: *Listeria monocytogenes*)

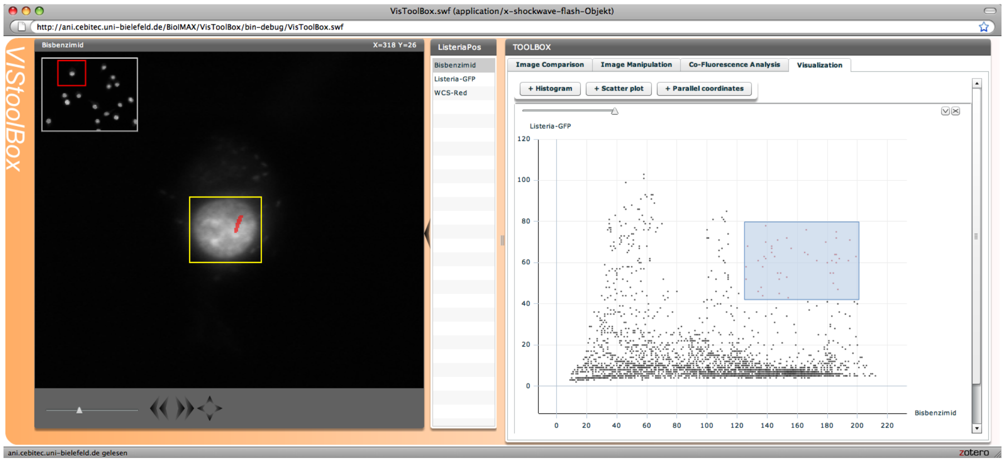


Figure 2: Detailed investigation of cell invasion on a single cell level. Here, the pixel values of a selected ROI of the nucleus and the *Listeria monocytogenes* channels are visualized in a scatter plot. The selection of those points in the plot showing high values in both channels reveals that *L. monocytogenes* are located within the nucleus (see red region in the selected cell). A full link&brush analysis is achieved due to the features of state-of-the-art web technology.

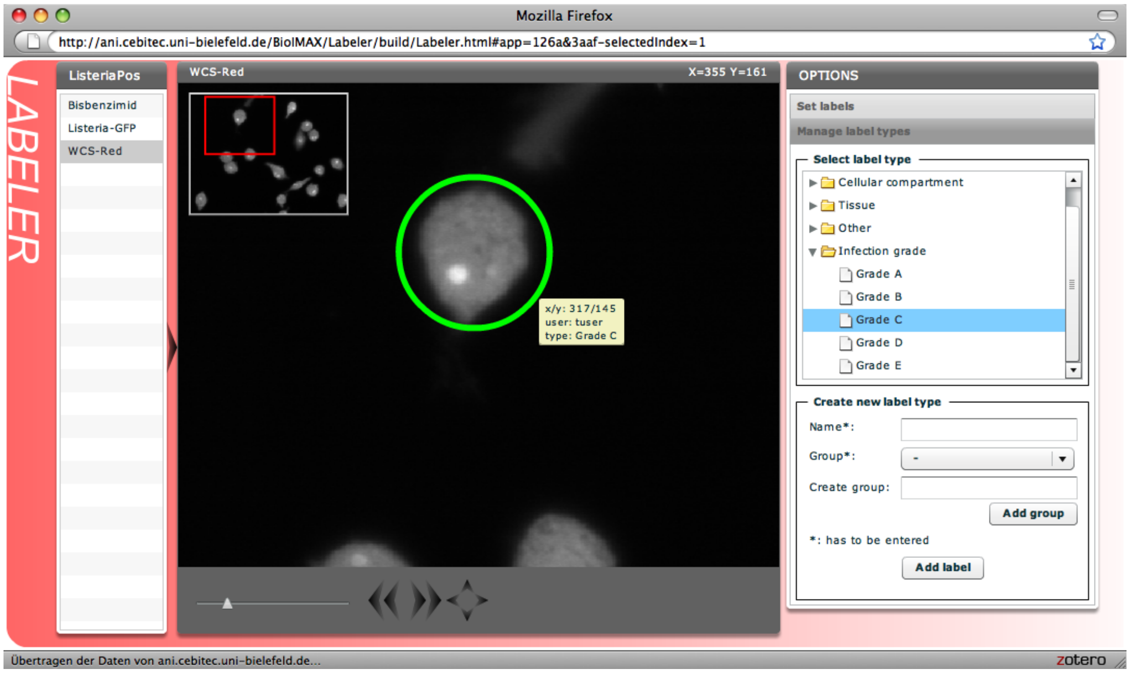


Figure 3: Annotating cells with different semantic categories, i.e., manually labeling cells regarding a specific infection grade. In this case, the resulting set of semantic labels are considered as a gold standard established by several human experts, in order to evaluate the precision of automatic segmentation or classification results.
