# Supplementary material for: BioIMAX: A Web 2.0 approach for easy exploratory and collaborative access to multivariate bioimage data
Source: BMC Bioinformatics. 2011 Jul 21;12:297. doi: 10.1186/1471-2105-12-297 (PMC3161928; doi:10.1186/1471-2105-12-297)
Supplement: Additional file 3 — Case study 2. [file 1471-2105-12-297-S3.PDF]

Case study 2

Investigating differences and similarities between Ion Mobility Spectrometry (IMS) image data and discussing specific image regions. IMS is a method to screen chemical compounds in mixtures like samples from the headspace of cell cultures or even more complex mixtures such as human breath. IMS samples are typically visualized as heat-map images and used to to detect and identify image regions as chemical compounds. In the following, some aspects of *BioIMAX* are illustrated, in order to investigate and compare a set of six patient samples.

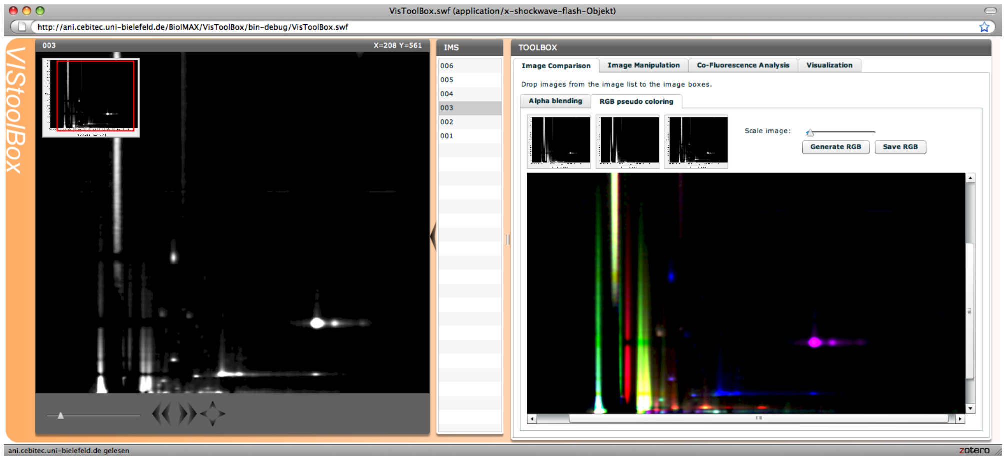

Figure 1: Simultaneously comparing three IMS images with the *RGB pseudo coloring* method, in order to detect differences or similarities between different samples.

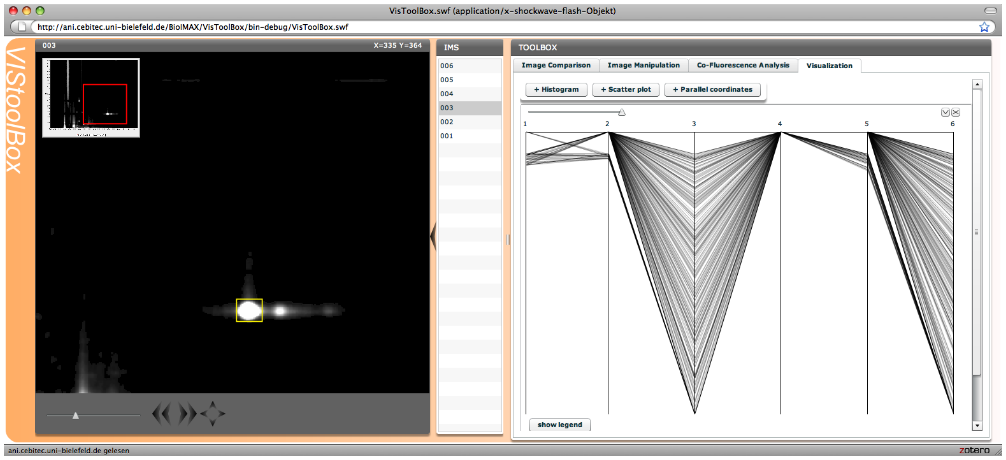

Figure 2: Investigating image regions of more than two or three images in one parallel coordinates plot (here regions of all six images). In a parallel coordinates plot, each polygonal line corresponds to a specific (x,y)-coordinate of the selected region, which intersects each of the equidistant axes at the point corresponding to the value of the respective image. With this plot it is possible to visualize the multidimensional characteristics of specific image regions, e.g., to visually identify groups or clusters of pixels.

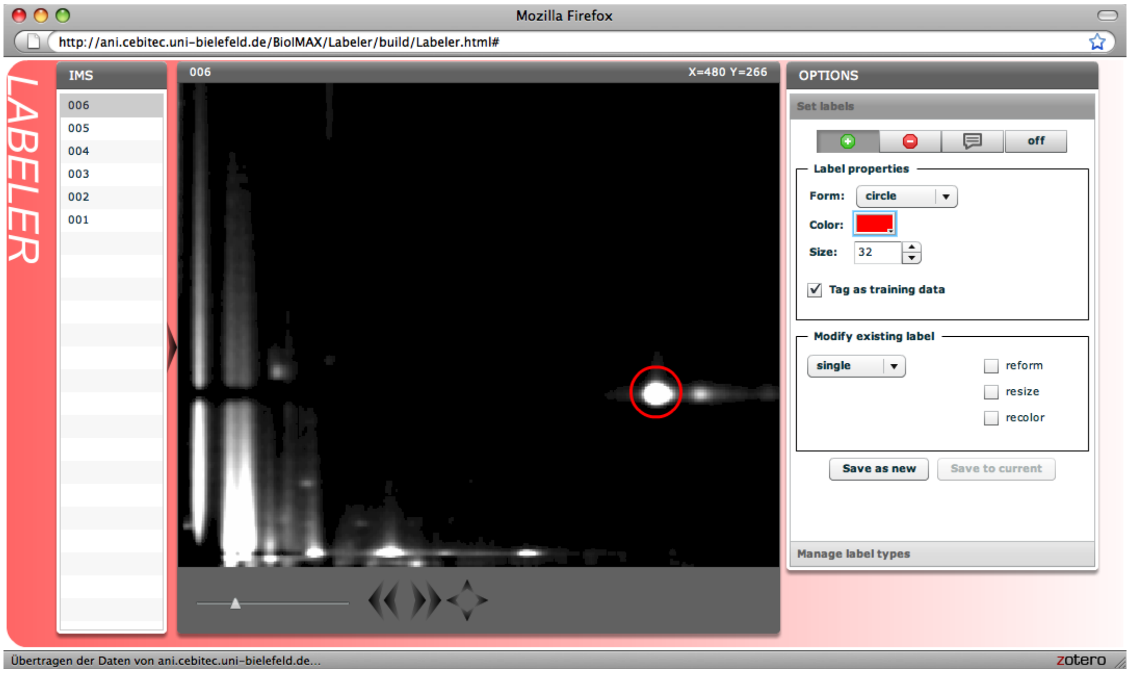

Figure 3: Discussing specific regions in the image with collaborating experts with the *Labeler* tool, e.g., regarding their trustworthiness or to avoid misinterpretations.
